# Risk of Atrial Fibrillation in Patients with Different Cancer Types in Taiwan

**DOI:** 10.3390/life14050621

**Published:** 2024-05-11

**Authors:** Kuang-Ming Liao, Chia-Hung Yu, Yu-Cih Wu, Jhi-Joung Wang, Fu-Wen Liang, Chung-Han Ho

**Affiliations:** 1 Department of Internal Medicine, Chi Mei Medical Center, Chiali, Tainan 722013, Taiwan; abc8870@yahoo.com.tw; 2 Department of Nursing, Min-Hwei Junior College of Health Care Management, Tainan 736302, Taiwan; 3 Department of Anesthesiology, Chi Mei Medical Center, Tainan 710402, Taiwan; dkntstar@hotmail.com (C.-H.Y.); 400002@mail.chimei.org.tw (J.-J.W.); 4 Department of Computer Science and Information Engineering, Southern Taiwan University of Science and Technology, Tainan 710301, Taiwan; 5 Department of Medical Research, Chi Mei Medical Center, Tainan 710402, Taiwan; cih830927@gmail.com; 6 Department of Public Health, College of Health Sciences, Kaohsiung Medical University, Kaohsiung 807378, Taiwan; 7 Department of Medical Research, Kaohsiung Medical University Hospital, Kaohsiung 807377, Taiwan; 8 Center for Big Data Research, Kaohsiung Medical University, Kaohsiung 807378, Taiwan; 9 Department of Information Management, Southern Taiwan University of Science and Technology, Tainan 710301, Taiwan; 10 Cancer Center, Taipei Municipal Wanfang Hospital, Taipei Medical University, Taipei 116079, Taiwan

**Keywords:** atrial fibrillation, cancer, competing risk, incidence rate, Taiwan Cancer Registry

## Abstract

Atrial fibrillation (AF) commonly occurs in approximately 2% of cancer patients, and the incidence of AF among cancer patients is greater than in the general population. This observational study presented the incidence risk of AF among cancer patients, including specific cancer types, using a population database. The Taiwan Cancer Registry was used to identify cancer patients between 2008 and 2017. The diagnosis of AF was based on the International Classification of Diseases codes (ICD-9-CM: 427.31 or ICD-10-CM: I48.0, I48.1, I48.2, and I48.91) in Taiwan national health insurance research datasets. The incidence of developing AF in the cancer population was calculated as the number of new-onset AF cases per person-year of follow-up during the study period. The overall incidence of AF among cancer patients was 50.99 per 100,000 person-years. Patients aged older than 65 years and males had higher AF incidence rates. Lung cancer males and esophageal cancer females showed the highest AF incidence risk (185.02 and 150.30 per 100,000 person-years, respectively). Our findings identified esophageal, lung, and gallbladder cancers as the top three cancers associated with a higher incidence of AF. Careful monitoring and management of patients with these cancers are crucial for early detection and intervention of AF.

## 1. Introduction

Atrial fibrillation (AF) is a common arrythmia characterized by irregular and often rapid heartbeats, and cancer patients have a higher risk of AF compared with the general population [1,2]. The current estimated prevalence of AF in adults is between 2% and 4%. It is expected to increase by roughly 2.3 times primarily due to an aging population and enhanced screening efforts for undetected AF. While increasing age is a major risk factor for AF, the rising incidence of other comorbidities also significantly contributes to its risk. Previously, the lifetime risk of developing AF was estimated to be one in four individuals; this estimate has recently been updated to one in three for individuals of European descent starting at the age of 55 years [3]. The prevalence of AF in cancer patients is approximately 20% [4]. The incidence of AF in cancer patients is approximately 30% and depends on the type of malignancy, surgical procedure, chemotherapy, and radiation therapy [5]. Previous studies have shown a correlation between cancer risk and the development of AF, and most have focused on colorectal or breast cancer [6,7,8,9,10,11]. A systematic review and meta-analysis revealed that patients with solid cancer had an increased risk of developing AF compared to patients without solid cancer [12]. The potential reasons for the increasing risk of AF among cancer patients included the detection of AF during cancer investigations, a possible association with systemic inflammation, and the utilization of antineoplastic drugs [13].

By using national databases, we demonstrated that the risk of AF was increased in all major cancer subtypes [10]. Another study conducted by Yun et al. [14] reported relationships between different cancer types and AF in the Korean National Health Insurance Service database between 2009 and 2016 and added to our understanding of AF risk in distinct cancer types. However, the study did not consider death as a competing risk, which may underestimate the incidence risk of AF. Therefore, we aimed to use Taiwanese national databases to analyze the risk of AF among patients with various types of cancer after adjusting for the risk of death and stratifying by sex, age and comorbidities.

## 2. Materials and Methods

### 2.1. Data Source

The Taiwan Cancer Registry (TCR) database was used in this study to estimate the incidence of AF among cancer patients. TCR data were collected for almost 97% of cancer patients in Taiwan. The TCR has collected the data of patients with newly diagnosed cancer in Taiwan since 1979, and the TCR central office uses standardized algorithms to validate the received data [15].

To estimate the incidence of AF, the TCR was linked with the National Health Insurance Research Database (NHIRD) to define patients who were diagnosed with AF and related comorbidities. The NHIRD was established according to the National Health Insurance (NHI) of Taiwan, which is a nationwide compulsory healthcare program that enrolls more than 99.6% of the population [16]. The database comprises detailed information regarding diagnostic codes, date of diagnosis, payments for consultations, and prescription details. The diagnosis codes in the NHIRD were based on the International Classification of Diseases, 9th Revision, and Clinical Modification (ICD-9-CM) codes for diagnoses and procedures before 2016, and the 10th Revision became effective after 2016.

### 2.2. Ethics Statement

This study was approved by the Institutional Review Board of Chi Mei Medical Centre (IRB: 11301-J02) and was conducted in compliance with the ethical standards and guidelines of the 2013 revision of the Declaration of Helsinki. Informed consent was also waived by the Institutional Review Board of Chi Mei Medical Centre due to the use of secondary databases and the absence of personal information in the study.

### 2.3. Study Participants and Outcomes

**Figure 1:**
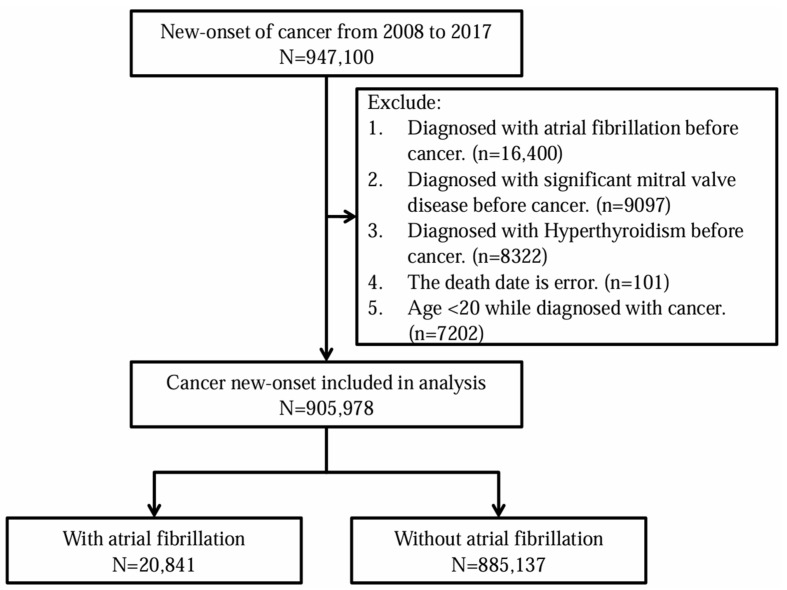
Flowchart of study subject selection.

Patients with cancer were identified using the TCR. The International Classification of Disease for Oncology, Third Edition (ICD-O-3), was used to identify patients with new-onset cancer corresponding to the period from 2008 to 2017 in the TCR database. The major outcome in this study was AF (ICD-CM-9:427.31; ICD-10: I48.0 I48.1 I48.2 I48.91). To estimate the incidence of new-onset AF among cancer patients, we excluded patients who were diagnosed with AF, hyperthyroidism, or important mitral valve disease before the date of cancer diagnosis. Additionally, some types of missing information were excluded to define a complete set of information for the study subjects. All study subjects were right-censored for follow-up until the first AF diagnosis, loss to follow-up, or the end of the study period, 31 December 2017. Figure 1 shows a flowchart of the study scheme.

The measurement confounding factors in this study included age, sex, and comorbidities. Age was defined as the age at cancer diagnosis. The comorbidities, including hypertension, diabetes mellitus, stroke, peripheral arterial occlusion disease (PAOD), heart failure, myocardial infarction, and end-stage renal disease (ESRD), were identified before one year of cancer diagnosis. To calculate the AF incidence risk among different cancer types, patients with different cancer types were classified by ICD-O-3. Subgroup analyses according to age and sex also assessed the risk of overall cancer and different cancer types. Details of the diagnosis codes of comorbidities and cancer types are shown in Supplementary Table S1.

### 2.4. Statistical Analysis

The baseline information, including age, sex, comorbidities, and death, between patients with AF and those without AF was estimated using Student’s *t*-test for continuous variables and Pearson’s Chi-square test for categorical variables. The incidence of developing AF in the cancer population was calculated as the number of new-onset AF cases per person-year of follow-up during the study period. The incidence rate of AF per 10,000 person-years at different patient baselines was calculated in this study. The Cox proportional hazards regression model was used to estimate the relative risk of AF for each variable of interest. Crude and adjusted hazard ratios with 95% confidence intervals are presented. Considering that death was a competing event during cancer treatment, we also performed a competing risk analysis to calculate the risk of AF using the Cox regression model with the Fine and Gray approach. For different types of cancer, the incidence rate of AF was also described for all study subjects, males, females, patients aged <65 years, and those aged more than 65 years. All analyses were conducted using SAS statistical software version 9.4 (SAS Institute, Inc., Cary, NC, USA). The statistical significance was set at a *p* value < 0.05.

## 3. Results

### 3.1. Baseline Characteristics

There were 905,978 patients enrolled in our study. These patients included 20,841 patients with cancer and AF and 885,137 patients with cancer without AF. As shown in Figure 1, there were 947,100 patients with cancer from 2008 to 2017, and 905,978 patients with cancer were included in analysis.

The overall mean age of the patients was 61.46 ± 14.90 years, 62.62% of the patients with cancer and AF were male, and 52.06% of the patients had cancer without AF. The two most common comorbidities were hypertension and diabetes mellitus in both groups. The baseline demographic data and comorbidities are presented in Table 1.

### 3.2. Risk of AF in Cancer Patients

Table 2 shows the incidence rates of AF and the crude and adjusted HRs for AF incidence in patients with cancer. The overall AF incidence rate was 64.29 per 10,000 person-years in patients diagnosed with cancer. During the follow-up period, 4786 (22.96%) cancer patients aged <65 years developed AF, and 16,055 cancer patients aged ≥65 years (77.04%) developed AF, with an adjusted HR of 4.67 (95% confidence interval [CI]: 4.51 to 4.84) compared with those aged <65 years after adjusting for sex and comorbidities, including hypertension, diabetes mellitus, myocardial infarction, stroke, PAOD, heart failure and ESRD. Among males, 13,051 (2.75%) had AF with an adjusted hazard ratio (HR) of 1.50 (95% confidence interval [CI]: 1.46 to 1.54) compared with females after adjusting for age, and comorbidities included hypertension, diabetes mellitus, myocardial infarction, stroke, PAOD, heart failure and ESRD. Patients with cancer and comorbidities, including hypertension, diabetes mellitus, myocardial infarction, stroke, PAOD, heart failure and ESRD, had an increased risk of AF compared with those without comorbidities.

### 3.3. Incidence of AF in Different Types of Cancer

Table 3 shows the incidence of AF in patients with different types of AF. The incidence of AF varied according to the type of cancer, and the incidence rate of AF varied according to cancer type. Compared with patients with different types of cancer, patients with esophageal cancer had the highest risk of AF, with an incidence rate of 155.94 per 10,000 person-years, and patients with thyroid cancer had the lowest risk of AF, with an incidence rate of 20.52 per 10,000 person-years.

Among solid cancers, the three highest risk factors for AF were esophageal cancer, lung cancer, and gallbladder and extrahepatic bile duct cancer, with incidence rates of 155.94, 137.18 and 108.11 per 10,000 person-years, respectively.

### 3.4. Incidence of AF in Different Types of Cancer Stratified by Sex

Table 4 shows the incidence of AF in different types of cancer stratified by sex. Among male patients with cancer, the three most common risk factors for AF were lung cancer, esophageal cancer, and gallbladder and extrahepatic bile duct cancer with incidence rates of 185.02, 156.42 and 116.88 per 10,000 person-years, respectively. Among female patients with cancer, the three most common risk factors for AF were esophagus cancer, skin cancer, and pancreatic cancer, with incidence rates of 150.3, 102.24, and 99.66 per 10,000 person-years, respectively.

### 3.5. Incidence of AF in Patients with Different Types of Cancer Stratified by Age

Table 5 shows the incidence of AF in different types of cancer stratified by age. Among patients aged <65 years, those with esophageal cancer had the highest risk of AF, followed by those with lung cancer and gallbladder and extrahepatic bile duct cancer, with incidence rates of 104.36, 58.35 and 39.38 per 10,000 person-years, respectively. Among patients aged ≥65 years, those with esophageal cancer had the highest risk of AF, followed by malignant neoplasm of thymus heart and mediastinum cancer and lung cancer, with incidence rates of 331.97, 307.63 and 231.57 per 10,000 person-years, respectively.

**Figure 2:**
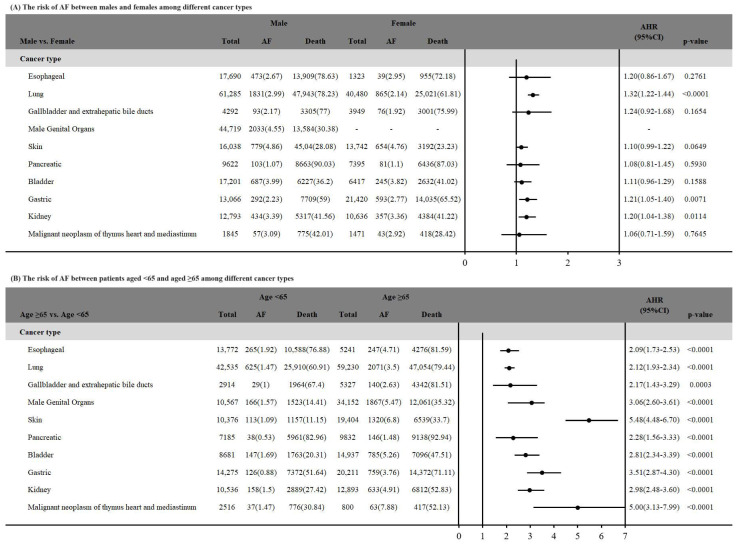
The risk of AF between among different cancer types by sex and age groups.

The impact of age and gender on AF across different cancer types, after considering death as a competing risk, is summarized in Figure 2. Our results indicate that male patients with lung cancer, gastric cancer, and kidney cancer demonstrate a statistically significant higher risk of AF compared to female patients. Additionally, patients aged 65 years and older show a significantly higher risk of developing AF in all cancer types compared with those aged under 65 years.

## 4. Discussion

Patients with cancer, particularly those receiving surgery for their condition, frequently experience AF. The heightened occurrence of AF among cancer patients may stem from existing medical conditions, the direct impact of the tumor, or complications arising from cancer-related surgical or pharmacological treatments. Inflammation could also be a significant factor connecting cancer with AF [17,18,19].

In our extensive population-based study, we discovered that patients with a history of cancer exhibited a greater risk of AF. The risk of AF varied depending on the type of cancer. Among various cancers, esophageal, lung, and gallbladder and extrahepatic bile duct cancer are the three most common cancer types associated with an increased risk of AF. Among males, the three most common risk factors for AF were lung cancer, esophageal cancer, and gallbladder and extrahepatic bile duct cancer. In females, esophagus cancer, skin cancer, and pancreatic cancer were the three most commonly associated with a greater risk of AF.

Identifying and managing AF is crucial in patients with cancer, as it represents a substantial additional health concern. Studies indicate that the risk of AF is notably greater in individuals with cancer [10,12,20]. A detailed meta-analysis reported a 47% increased likelihood of AF among these patients (odds ratio: 1.47; 95% CI: 1.31 to 1.66) [12]. Additionally, research from Denmark revealed that cancer patients had a 1.4-fold greater incidence of AF than individuals in the broader population [10]. Even those cancer patients who did not undergo active treatment exhibited a 20% greater risk of AF (odds ratio: 1.19; 95% CI: 1.02 to 1.38) [20]. Our own research indicated an AF occurrence rate of 64.29 per 10,000 person-years in individuals diagnosed with cancer.

In our study of patients with cancer, we found that the incidence of AF increased with age. Compared with female patients, male patients exhibited a greater risk of AF. Additionally, the presence of several comorbidities, including hypertension, diabetes mellitus, stroke, PAOD, heart failure, myocardial infarction, and ESRD, was associated with an increased risk of AF among cancer patients. Ay et al. [21] reported that the incidence of AF increased with age and was greater in the male population than in the female population among cancer patients. In a study by Zubair et al. [22], it was also demonstrated that many comorbidities are associated with AF in cancer patients, including coronary artery disease, obstructive sleep apnea, congestive heart failure, valvular disease, chronic pulmonary disease, hypertension, diabetes mellitus, hypothyroidism, renal failure, obesity, and collagen vascular disease. However, our study differs from that of Ay et al. [21]. First, we showed the incidence data of AF in cancer patients and presented the incidence rate. Their study showed the prevalence of AF in cancer patients and odds ratios (ORs) in cancer subgroups. Second, in our study of the incidence rate of AF in cancer patients regardless of sex, the three most common three highest risk factors for AF were esophageal, lung, and gallbladder and extrahepatic bile duct cancer. In their study, the prevalence of AF was highest in lung cancer, followed by prostate cancer, Hodgkin’s lymphoma, non-Hodgkin’s lymphoma and leukemia in cancer patients aged less than 65 years. In patients aged from 65 to 80, the prevalence of AF was highest in lung cancer followed by prostate cancer, non-Hodgkin’s lymphoma, leukemia, multiple myeloma and Hodgkin’s lymphoma [17,23]. 

Our study found sex differences in AF incidence, with male cancer patients having a 1.50-fold higher risk of AF compared to females (HR: 1.50, 95% CI: 1.46–1.54, *p* < 0.0001). The higher prevalence of smoking and alcohol consumption in males compared to females in Taiwan [24,25] may be the potential reason that male cancer patients had a higher risk of AF, as both smoking and drinking are common risk factors for AF and cancer [26,27]. The synergistic effect of these risk factors may further increase the incidence of cancer and AF in males. However, it is worth noting that sex differences in AF risk may also be influenced by hormonal levels [28,29]. Therefore, the impact of sex differences on AF incidence may involve multiple factors and requires further research.

Our study revealed that the incidence rate of AF in cancer patients was 64.29 per 10,000 person-years, aligning with previous research that reported an AF incidence of 66 per 10,000 person-years in patients with cancer [14]. Among solid tumors, lung and esophageal cancers were found to be significantly associated with an increased risk of AF. Unlike their study [14], a competing risk analysis was used to assess the risk of AF in patients with cancer in our study. Incidence analysis is widely used in cancer research to explore clinical questions, including the incidence of AF. The detection of AF is contingent upon patients being alive, highlighting the necessity of accounting for patients’ survival status. As treatments and prognoses for various cancers improve, the importance of outcomes other than death is increasingly recognized. In studies focusing on the incidence of AF among cancer patients, competing risks present a challenge, as patients may encounter events that preclude the onset of the primary outcome of interest, AF [30].

Differences in AF prevalence among various cancers may be attributed to different cancer incidence rates and patients’ risk factors across countries. In Taiwan, lung cancer is the most common cancer, primarily due to smoking, which also increases the risk for AF. Globally, esophageal cancer is the eighth most prevalent cancer, with age-standardized incidence rates of 9.3 per 100,000 in men and 3.6 per 100,000 in women according to GLOBOCAN 2020 [31]. It is most frequently diagnosed in Central and East Asia. In Taiwan, the age-standardized incidence rate of esophageal cancer has significantly increased from 4.88 per 100,000 in 1985 to 23.83 per 100,000 in 2019 [32].

The primary risk factors for esophageal squamous cell carcinoma in Taiwan are smoking, alcohol consumption, and betel nut chewing, which are together responsible for 83.7% of the attributable cases of this cancer [33]. The synergistic effect of alcohol and tobacco significantly escalates the risk of developing esophageal squamous cell carcinoma [34]. The high prevalence of these combined risk factors in Taiwanese men may lead to a greater incidence of AF associated with esophageal cancer in men than from smoking or alcohol consumption alone.

Moreover, the occurrence of AF may affect the oncologic management and quality of life among cancer patients. Chen et al. showed that both cancer diagnosed after incident AF (HR: 7.77, 95% CI: 7.45–8.11) and AF diagnosed after incident cancer (HR: 2.55, 95% CI: 2.47–2.63) were associated with all-cause mortality, but the strength of the association varied by cancer type [35]. Compared to patients with baseline AF, those with new-onset AF in the context of malignancy had a two-fold increased risk of thromboembolism and a six-fold increased risk of heart failure, highlighting the importance of prompt recognition and treatment of AF during cancer treatment [6,36]. Previous studies also indicated that patients with AF had lower quality [37,38]. Therefore, future research should explore the impact of AF on oncologic management, including its effect on treatment decisions, hospital admissions, and quality of life among cancer patients.

### 4.1. Limitations

Our study has several limitations that need to be acknowledged. First, the claims database lacked specific data on how patients responded to treatment, the strategies employed in treatment, and biomarker information. There are no data regarding the type of oncologic treatment per cancer type (radiation, chemotherapy, immunotherapy, and surgery), which is clinically meaningful and might have impacted the incidence of post-surgical AF among certain types of malignancy. Additionally, due to the incomplete treatment records for all cancer types in the TCR, as well as the variation in treatment modalities for different types of cancer, the treatment type was not included in our analysis. Future studies should focus on specific cancer subgroups to investigate the impact of comorbidities and treatment modalities on the incidence of AF.

Secondly, there was a possibility of misclassification of diagnoses based on the claims database. However, it is important to note that the diagnosis of cancer in TCR, known for its high-quality cancer registration system [39], was established using the ICD-O-3. Moreover, under the scheme of National Health Insurance (NHI) in Taiwan, cancer patients have access to catastrophic illness certification, exempting them from specific NHI charges and copayments for healthcare visits. Every request for this certification undergoes expert review, ensuring a high level of diagnostic precision [16]. Thus, the potential for misclassification bias is minimized. 

Furthermore, the reliability of AF diagnoses within Taiwan’s National Health Insurance Research Database (NHIRD) has been confirmed to be highly accurate [40,41,42]. Moreover, detection bias may also have affected our results. Cancer patients undergo frequent medical check-ups, and the chances of discovering asymptomatic AF are greater in these individuals than in those who do not have frequent medical appointments. Furthermore, the scope of our study was constrained by its reliance on data from a single country, lacking details on different racial and socioeconomic backgrounds. Our study was conducted exclusively within the Taiwanese population; hence, any generalization of the findings to other racial groups should be approached with caution. Another limitation of our study was that we did not investigate the roles of metabolic syndrome, chronic obstructive pulmonary disease, obesity, sleep apnea, alcohol consumption or valvular heart disease as risk factors. Chronic obstructive pulmonary disease is associated with AF due to a range of factors, including hypoxia, chronic inflammation, oxidative stress, hypercapnia, pulmonary hypertension, diastolic dysfunction, and changes in atrial size resulting from altered respiratory physiology. Additionally, the impact of respiratory medications also contributes to the risk of AF in individuals with chronic obstructive pulmonary disease. Similarly, valvular heart disease is commonly linked with AF, particularly in cases involving mitral stenosis, mitral regurgitation, or tricuspid regurgitation. Such conditions lead to extensive remodeling of the atria, which facilitates the maintenance of atrial fibrillation.

### 4.2. Strengths

The main strength of our study is highlighted by the inclusion of the most extensive patient cohort across a wide array of cancer types, revealing a link between the occurrence of cancer and AF incidence and the high accuracy of diagnosing cancer and AF. We detailed the AF incidence rates for our entire cohort, accounting for various comorbid conditions, and analyzed the data based on sex for individuals younger than 65 years and for those aged 65 years and older. Since recent clinical guidelines advocate for routine AF screening in patients aged 65 years and older [3], our results contribute valuable information supporting the consideration of routine AF screening for individuals within this age group. Furthermore, we conducted a competing risk analysis using the Cox regression model, incorporating the Fine and Gray method, to evaluate AF risk. This approach could reduce the limitation of individuals potentially developing AF but being censored due to death.

### 4.3. Prospective Study

According to our finding and the above limitations, several critical issues require further prospective study. There is a need to explore the management of AF patients, specifically how various approaches to anticoagulation and rate control differ across countries and may lead to varying stroke risks. Additionally, the types of oncologic treatments—such as radiation, chemotherapy, immunotherapy, and surgery—vary per cancer type and have significant clinical implications. These treatments could influence the incidence of post-treatment AF in certain malignancies and warrant further investigation. Variations in AF incidence across different cancers might be attributed to differences in cancer rates and distinct risk factors prevalent in different patients. Investigating the influence of factors like metabolic syndrome, chronic obstructive pulmonary disease, obesity, sleep apnea, alcohol consumption, and valvular heart disease, which are also risk factors for AF, is essential. In the cancer population, the risks of venous thromboembolic disease and arterial thromboembolism, including stroke, generally increase across most cancer types. Moreover, AF itself is an independent risk factor for stroke. The relationship between cancer patients with AF and their risk of stroke or bleeding outcomes remains unclear. Furthermore, data on stroke and bleeding incidence reported in the limited number of cancer patients enrolled in clinical trials show varying results. Therefore, data on the distribution of stroke and bleeding risk among cancer patients with AF are notably insufficient. All these aspects necessitate further research.

## 5. Conclusions

In summary, our findings reveal that the incidence of AF varies among patients with cancer, indicating a significantly increased risk of AF across different comorbidities and cancer types. The increasing prevalence within this demographic introduces new and unique challenges for the field of cardiology. Consequently, additional research is imperative to inform treatment strategies and understand the causal relationships between various cancers, their treatments, and the onset of AF. Furthermore, future studies should not only explore the impact of AF on the prognosis of cancer patients but also consider the distinctions in overall management between individuals with cancer and the general population. Finally, our findings highlight the importance of multidisciplinary care for patients with both cancer and AF. Collaborative efforts between oncologists, cardiologists, and other healthcare providers are essential to optimize the management of these complex patients and improve their overall quality of care.

## Figures and Tables

**Table 1:** Demographic characteristics and comorbidities of cancer patients with and without atrial fibrillation.

	Overall (N = 905,978)	With Atrial Fibrillation (N = 20,841)	Without Atrial Fibrillation (N = 885,137)	*p* Value
Age, years (mean ± SD)	61.46 ± 14.90	72.54 ± 10.89	61.20 ± 14.89	<0.0001
Age stratification (%)				
<65	520,914 (57.50)	4786 (22.96)	516,128 (58.31)	<0.0001
≥65	385,064 (42.50)	16,055 (77.04)	369,009 (41.69)	
Sex (%)				
Male	473,855 (52.30)	13,051 (62.62)	460,804 (52.06)	<0.0001
Female	432,123 (47.70)	7790 (37.38)	424,333 (47.94)	
Comorbidities (%)				
Hypertension	327,009 (36.09)	12,326 (59.14)	314,683 (35.55)	<0.0001
Diabetes mellitus	172,174 (19.00)	5366 (25.75)	166,808 (18.85)	<0.0001
Stroke	65,119 (7.19)	2694 (12.93)	62,425 (7.05)	<0.0001
PAOD	15,785 (1.74)	687 (3.30)	15,098 (1.71)	<0.0001
Heart failure	33,594 (3.71)	2335 (11.20)	31,259 (3.53)	<0.0001
Myocardial infarction	7486 (0.83)	348 (1.67)	7138 (0.81)	<0.0001
ESRD	3176 (0.35)	81 (0.39)	3095 (0.35)	0.3465
Death	405,115 (44.72)	11,727 (56.27)	393,388 (44.44)	<0.0001

PAOD: peripheral arterial occlusion disease; ESRD: end-stage renal disease.

**Table 2:** The risk of atrial fibrillation in cancer patients.

Characteristics	Patients	Event of AF	PYs	Incidence Rate	Crude HR (95% C.I.)	*p*	Adjusted HR (95% C.I.)	*p*
Overall	905,978	20,841 (2.3)	3,241,527	64.29	-		-	
Age								
<65	520,914	4786 (22.96)	2,169,483.20	22.06	Ref.		Ref.	
≥65	385,064	16,055 (77.04)	1,072,043.80	149.76	6.33 (6.13–6.54)	<0.0001	4.67 (4.51–4.84)	<0.0001
Sex								
Male	473,855	13,051 (2.75)	1,467,721.58	88.92	1.90 (1.85–1.96)	<0.0001	1.50 (1.46–1.54)	<0.0001
Female	432,123	7790 (1.80)	1,773,805.42	43.92	Ref.		Ref.	
Comorbidities								
Hypertension								
Yes	327,009	12,326 (3.77)	1,017,778.61	121.11	3.03 (2.94–3.11)	<0.0001	1.57 (1.52–1.62)	<0.0001
No	578,969	8515 (1.47)	2,223,748.39	38.29	Ref.		Ref.	
Diabetes mellitus					9			
Yes	172,174	5366 (3.12)	502,778.54	106.73	1.78 (1.73–1.84)	<0.0001	1.02 (0.99–1.06)	0.1423
No	733,804	15,475 (2.11)	2,738,748.47	56.5	Ref.		Ref.	
Myocardial infarction								
Yes	7486	348 (4.65)	17,420.19	199.77	2.83 (2.55–3.15)	<0.0001	1.26 (1.13–1.40)	<0.0001
No	898,492	20,493 (2.28)	3,224,106.81	63.56	Ref.		Ref.	
Stroke								
Yes	65,119	2694 (4.14)	158,914.73	169.52	2.62 (2.52–2.73)	<0.0001	1.19 (1.14–1.24)	<0.0001
No	840,859	18,147 (2.16)	3,082,612.27	58.87	Ref.		Ref.	
PAOD								
Yes	15,785	687 (4.35)	43,905.58	156.47	2.34 (2.17–2.52)	<0.0001	1.22 (1.13–1.31)	<0.0001
No	890,193	20,154 (2.26)	3,197,621.42	63.03	Ref.		Ref.	
Heart failure								
Yes	33,594	2335 (6.95)	77,674.41	300.61	4.65 (4.45–4.85)	<0.0001	2.31 (2.21–2.42)	<0.0001
No	872,384	18,506 (2.12)	3,163,852.59	58.49	Ref.		Ref.	
ESRD								
Yes	3176	81 (2.55)	4084.2	198.33	2.13 (1.71–2.65)	<0.0001	1.31 (1.05–1.63)	0.0159
No	902,802	20,760 (2.30)	3,237,442.8	64.12	Ref.		Ref.	

AF: atrial fibrillation; PYs: person years; PAOD: peripheral arterial occlusion disease; ESRD: end-stage renal disease.

**Table 3:** The risk of atrial fibrillation in patients with different cancer types.

Cancer Type	Patients	With AF	Without AF	PYs	Incidence Rate
Esophageal	19,013	512 (2.69)	18,501 (97.31)	32,833.31	155.94
Lung	101,765	2696 (2.65)	99,069 (97.35)	196,536.87	137.18
Gallbladder and extrahepatic bile ducts	8241	169 (2.05)	8072 (97.95)	15,632.73	108.11
Male genital organs	44,720	2033 (4.55)	42,687 (95.45)	190,265.33	106.85
Skin	29,780	1433 (4.81)	28,347 (95.19)	134,729.49	106.36
Pancreatic	17,017	184 (1.08)	16,833 (98.92)	17,428.05	105.58
Bladder	23,618	932 (3.95)	22,686 (96.05)	95,376.87	97.72
Gastric	34,486	885 (2.57)	33,601 (97.43)	94,212.39	93.94
Kidney	23,429	791 (3.38)	22,638 (96.62)	87,304.02	90.6
Malignant neoplasm of thymus heart and mediastinum	3316	100 (3.02)	3216 (96.98)	12,096.85	82.67
Liver	101,273	1920 (1.9)	99,353 (98.1)	236,491.44	81.19
Colorectal	137,841	3976 (2.88)	133,865 (97.12)	526,792.81	75.48
Malignant neoplasm of connective and other soft tissue	4594	122 (2.66)	4472 (97.34)	17,665.49	69.06
Small intestine	3566	82 (2.3)	3484 (97.7)	12,087.33	67.84
Peritoneum	1634	25 (1.53)	1609 (98.47)	4651.76	53.74
Eye	771	19 (2.46)	752 (97.54)	3730.35	50.93
CNS cancer	6916	94 (1.36)	6822 (98.64)	18,637.03	50.44
Head and neck	84,313	1381 (1.64)	82,932 (98.36)	333,474.91	41.41
Bone	1116	18 (1.61)	1098 (98.39)	4490.1	40.09
Breast	115,996	1269 (1.09)	114,727 (98.91)	581,233.59	21.83
Gynecologic	74,669	820 (1.1)	73,849 (98.9)	383,875	21.36
Thyroid	25,160	263 (1.05)	24,897 (98.95)	128,149.54	20.52
Others	42,745	1117 (2.61)	41,628 (97.39)	113,838	98.12

AF: atrial fibrillation; PYs: person years.

**Table 4:** Incidence of atrial fibrillation in cancer patients stratified by sex.

	Male				Female			
Cancer Type	Patients	With AF	PYs	Incidence Rate	Patients	With AF	PYs	Incidence Rate
Lung	61,285	1831 (2.99)	98,963.86	185.02	40,480	865 (2.14)	97,573.01	88.65
Esophageal	17,690	473 (2.67)	30,238.53	156.42	1323	39 (2.95)	2594.78	150.3
Gallbladder and extrahepatic bile ducts	4292	93 (2.17)	7956.93	116.88	3949	76 (1.92)	7675.8	99.01
Pancreatic	9622	103 (1.07)	9300.22	110.75	7395	81 (1.1)	8127.83	99.66
Skin	16,038	779 (4.86)	70,761.82	110.09	13,742	654 (4.76)	63,967.66	102.24
Breast	443	21 (4.74)	1936.13	108.46	115,553	1248 (1.08)	579,297.45	21.54
Male genital organs	44,719	2033 (4.55)	190,259.08	106.85	-	-	-	-
Gastric	21,420	593 (2.77)	55,531.75	106.79	13,066	292 (2.23)	38,680.64	75.49
Bladder	17,201	687 (3.99)	70,660.26	97.23	6417	245 (3.82)	24,716.61	99.12
Peritoneum	672	17 (2.53)	1843.59	92.21	962	8 (0.83)	2808.18	28.49
Malignant neoplasm of thymus heart and mediastinum	1845	57 (3.09)	6190.35	92.08	1471	43 (2.92)	5906.5	72.8
Kidney	12,793	434 (3.39)	47,401.71	91.56	10,636	357 (3.36)	39,902.31	89.47
Connective and other soft tissue	2679	83 (3.1)	10,055.64	82.54	1915	39 (2.04)	7609.85	51.25
Colorectal	80,750	2507 (3.1)	304,351.01	82.37	57,091	1469 (2.57)	222,441.81	66.04
Small intestine	2116	54 (2.55)	6901.49	78.24	1450	28 (1.93)	5185.83	53.99
Liver	71,332	1251 (1.75)	164,903.71	75.86	29,941	669 (2.23)	71,587.74	93.45
Eye	403	12 (2.98)	1920.63	62.48	368	7 (1.9)	1809.71	38.68
CNS cancer	3862	46 (1.19)	9904.06	46.45	3054	48 (1.57)	8732.98	54.96
Head and neck	73,100	1192 (1.63)	284,135.66	41.95	11,213	189 (1.69)	49,339.26	38.31
Bone	633	9 (1.42)	2513.75	35.8	483	9 (1.86)	1976.35	45.54
Thyroid	6055	92 (1.52)	29,120.33	31.59	19,105	171 (0.9)	99,029.21	17.27
Gynecologic	0	0 (0)	0	0	74,669	820 (1.1)	383,875	21.36
Others	24,905	684 (2.75)	62,871.08	108.79	17,840	433 (2.43)	50,966.92	84.96

AF: atrial fibrillation; PYs: person years.

**Table 5:** Incidence of atrial fibrillation in cancer patients stratified by age group.

	<65				≥65			
Cancer Type	Patients	With AF	PYs	Incidence Rate	Patients	With AF	PYs	Incidence Rate
Esophageal	13,772	265 (1.92)	25,392.91	104.36	5241	247 (4.71)	7440.41	331.97
Lung	42,535	625 (1.47)	107,103.09	58.35	59,230	2071 (3.5)	89,433.78	231.57
Gallbladder and extrahepatic bile ducts	2914	29 (1.00)	7363.95	39.38	5327	140 (2.63)	8268.78	169.31
Pancreatic	7185	38 (0.53)	10,066.16	37.75	9832	146 (1.48)	7361.89	198.32
Malignant neoplasm of thymus heart and mediastinum	2516	37 (1.47)	10,048.92	36.82	800	63 (7.88)	2047.93	307.63
Bladder	8681	147 (1.69)	42,650.85	34.47	14,937	785 (5.26)	52,726.03	148.88
Kidney	10,536	158 (1.50)	46,672.57	33.85	12,893	633 (4.91)	40,631.45	155.79
Liver	50,358	444 (0.88)	133,665.53	33.22	50,915	1476 (2.90)	102,825.92	143.54
Male genital organs	10,567	166 (1.57)	51,015.16	32.54	34,152	1867 (5.47)	139,243.91	134.08
Bone	862.00	11 (1.28)	3821.1	28.79	254	7 (2.76)	669.00	104.63
Gastric	14,275	126 (0.88)	48,919.06	25.76	20,211	759 (3.76)	45,293.33	167.57
Small intestine	1907	20 (1.05)	7846.47	25.49	1659	62 (3.74)	4240.86	146.20
Connective and other soft tissue	2959	33 (1.12)	12,990.8	25.4	1635	89 (5.44)	4674.69	190.39
Colorectal	68,253	740 (1.08)	293,692.62	25.2	69,588	3236 (4.65)	233,100.19	138.82
Head and neck	66,490	658 (0.99)	277,514.73	23.71	17,823	723 (4.06)	55,960.18	129.20
CNS cancer	4649	33 (0.71)	15,282.01	21.59	2267	61 (2.69)	3355.03	181.82
Skin	10,376	113 (1.09)	54,561.16	20.71	19,404	1320 (6.8)	80,168.33	164.65
Peritoneum	1040	6 (0.58)	3521.02	17.04	594	19 (3.20)	1130.75	168.03
Eye	468	3 (0.64)	2376.56	12.62	303	16 (5.28)	1353.79	118.19
Thyroid	22,002	114 (0.52)	115,765.6	9.85	3158	149 (4.72)	12,383.93	120.32
Breast	95,018	473 (0.50)	490,987.65	9.63	20,978	796 (3.79)	90,245.93	88.20
Gynecologic	61,535	272 (0.44)	330,285.3	8.24	13,134	548 (4.17)	53,589.7	102.26
Others	22,016	275 (1.25)	77,940.01	35.28	20,729	842 (4.06)	35,898	234.55

AF: atrial fibrillation; PYs: person years.

## Data Availability

The data sources are the Taiwan Nation Health Insurance Database and Taiwan Cancer Registry. The data are available with permission from the Taiwan Health and Welfare Data Science Center (https://dep.mohw.gov.tw/DOS/cp-5119-59201-113.html, accessed on 20 March 2024). Restrictions apply to the availability of these data, which were used under license for this study.

## Supplementary Materials

The following supporting information can be downloaded at: https://www.mdpi.com/article/10.3390/life14050621/s1, Table S1: International Classification of Diseases for Oncology, 3rd Edition (ICD-O-3) for cancers and International Classification of Diseases, Ninth Revision, Clinical Modification (ICD-9-CM) and Tenth Revision, Clinical Modification (ICD-10-CM) Diagnosis for comorbidities.
