# Supplementary material for: Risk of Atrial Fibrillation in Patients with Different Cancer Types in Taiwan
Source: Life (Basel). 2024 May 11;14(5):621. doi: 10.3390/life14050621 (PMC11122475; doi:10.3390/life14050621)
Supplement: Supplementary file 1 [file life-14-00621-s001.zip › life-2973374-supplementary.pdf]

Supplemental Table S1. International Classification of Diseases for Oncology, 3rd Edition (ICD-O-3) for cancers and International Classification of Diseases, Ninth Revision, Clinical Modification (ICD-9-CM) and Ten Revision, Clinical Modification (ICD-10-CM) Diagnosis for comorbidities.

	ICD-3-O	ICD-9-CM	ICD-10-CM
**Cancer**	C00-C80		
Esophageal	C15		
Lung	C33-C34		
Gallbladder and extrahepatic bile ducts	C23-C24		
Male Genital Organs	C60-C63		
Skin	C44		
Pancreatic	C25		
Bladder	C67		
Gastric	C16		
Kidney	C64-C66, C68		
Malignant neoplasm of thymus heart and mediastinum	C37-C38		
Liver	C22		
Colorectal	C18-C21		
Malignant neoplasm of connective and other soft tissue	C47, C49		
Small intestine	C17		
Peritoneum	C26, C48		
Eye	C69		
CNS cancer	C70-C72		
Head and Neck	C00-C14		
Bone	C40-C41		
Breast	C50		
Gynecologic	C51-C58		
Thyroid	C73		
**Outcome**			
Atrial fibrillation		427.31	I48.0-I48.2, I48.91

	ICD-3-O	ICD-9-CM	ICD-10-CM
**Comorbidity**			
Hypertension		401-405	I10-I15
Diabetes		250	E10-E14
Stroke		430-438, 362.34	G45-G46, I60-I69, H34.0
Peripheral arterial occlusion disease (PAOD)		093.0, 437.3, 437.1, 440, 441, 443.1-443.9, 444.22, 444.8, 447.1, 447.8, 447.9, 471, 557.1, 557.9	I70, I71, I73.1, I73.8, I73.9, I77.1, I79.0, I79.2, K55.1, K55.8, K55.9, Z95.8, Z95.9
Heart failure		428, 435.4-435.9, 398.91, 402.01, 402.11, 402.91, 404.01, 404.03, 404.11, 404.13, 404.91, 404.93	I43, I50, I09.9, I11.0, I13.0, I13.2, I25.5, I42.0, I42.5-I42.9, P29.0
Myocardial infarction		410 412	I21 I22 I25.2
End stage renal disease (ESRD)		585.5, 585.6	N18.6
